# Vaping cartridge heating element compositions and evidence of high temperatures

**DOI:** 10.1371/journal.pone.0240613

**Published:** 2020-10-19

**Authors:** Jeff Wagner, Wenhao Chen, Gordon Vrdoljak

**Affiliations:** 1 Outdoor Air Quality Section, Environmental Health Laboratory Branch, California Department of Public Health, Richmond, California, United States of America; 2 Indoor Air Quality Section, Environmental Health Laboratory Branch, California Department of Public Health, Richmond, California, United States of America; 3 Cannabis Testing Section, Food and Drug Laboratory Branch, California Department of Public Health, Richmond, California, United States of America; Medical University of South Carolina, UNITED STATES

## Abstract

**Background:**

Identifying the functional materials inside vaping devices can help inform efforts to understand risk. While nicotine E-cigarette components and metals have been characterized in several previous studies, the internal component compositions of tetrahydrocannabinol (THC) cartridge designs are not as well known. The 2019–20 e-cigarette or vaping product use associated lung injury (EVALI) outbreak has been associated with THC devices containing vitamin E acetate (VEA), possibly mediated by chemical reactions with internal cartridge components and high filament temperatures.

**Methods:**

We investigate the composition and internal components of 2019 EVALI patient-associated THC vaping devices compared to other THC and nicotine devices from 2016–19, specifically the metal, ceramic, and polymer components likely to be exposed to heat. To do this, we have disassembled forty-eight components from eight used and unused vaping devices under a microscope and analyzed them using X-ray fluorescence, scanning electron microscopy, and Fourier-transform infrared micro-spectroscopy.

**Conclusions:**

The two THC cartridges used by EVALI patients exhibited evidence of localized high temperatures, including charring of the ceramic heating elements and damaged wire surfaces. The newer THC cartridges possessed more ceramic and polymer insulation than older THC or nicotine devices. The combination of ceramics, metals, and high temperatures in newer THC cartridges is consistent with conditions hypothesized to produce VEA reactions during vaping. Nickel and chromium components were detected in all devices, and others contained copper, lead, tin, gold, silicon-rich rubbers, or fluorinated microplastics. These components have the potential to thermally degrade and volatilize if heated sufficiently. These findings do not imply that harmful exposures would occur under all usage conditions, and are most relevant to harm reduction efforts based on avoiding higher internal temperatures. This study was limited to a small sample of cartridges obtained from investigations. Future work should test more device types and internal temperatures under controlled usage conditions.

## Introduction

Identifying the functional materials inside vaping devices can help inform efforts to understand risk. An outbreak of e-cigarette or vaping product use associated lung injury (EVALI) in 2019–20 resulted in hospital admissions and deaths. Vitamin E acetate (VEA) inside tetrahydrocannabinol (THC) vaping devices was identified as the most likely indicator of injury [1, 2]. Acute lung injury has been reported in EVALI lung biopsies and autopsies [3, 4], and it has been hypothesized that VEA may have chemically reacted to create toxic ketene (ethenone) in the presence of high temperatures [5]. Because chemical reactions can be catalyzed on the surfaces of ceramics or metals such as nickel (Ni) or cobalt (Co) [6, 7], any ceramics or metals present inside the vaping devices could further enhance these reactions [8].

A variety of nicotine E-cigarette cartridges and pods have been characterized by determining their design components [9], testing their heating coil temperature profiles [10, 11], and measuring metal content of their e-liquids and airborne emissions [12–16]. To our knowledge, little comparable information exists in the peer reviewed literature for THC vaping devices [17, 18].

Scanning electron microscopy with energy dispersive X-ray spectroscopy (SEM-EDS) and Fourier-transform infrared (FTIR) micro-spectroscopy are two methods previously used to infer potential environmental pathways from microscale elemental chemistry and morphology of consumer products [19–21]. Williams et al [22] used SEM-EDS to characterize nicotine device components. Alternatively, portable/handheld X-ray fluorescence (XRF) units can obtain heavy metal concentrations from device surfaces more rapidly and with little sample preparation, though they do not provide detailed surface imaging.

In this paper, we determined the composition and internal components of EVALI patient-associated THC vaping devices compared to other THC and nicotine devices from 2016–19, specifically the metal, ceramic, and polymer components likely to be exposed to heat. To do this, we have disassembled forty-eight components from eight used and unused THC and nicotine vaping devices under a microscope and analyzed them using portable XRF, SEM-EDS, and limited FTIR micro-spectroscopy.

## Materials and methods

Six THC vape cartridges and two nicotine e-cigarette devices were characterized (Table 1). Two of the tested THC cartridges were used by EVALI patients and were obtained as part of California’s EVALI response in 2019 [2]. These products were designated “CCell #1” and “TKO” based on the etchings on their respective battery contact end caps, but additional information about their models or authenticity is unknown. Two other unused, filled cartridges were obtained as part of routine cannabis product testing and surveillance investigations in 2018 (no identifiable product markings, designated “unknown #1” and “unknown #2”). The liquids from these four cartridges were previously extracted for separate analyses. In addition, four products purchased new online between 2016–19 were analyzed for comparison purposes: two empty THC vaping products (CCell TH2 Oil Cartridge and Linx Hermes atomizer) and two nicotine devices (a filled JUUL pod and an eGo-CE6 coil head). The JUUL pod was used in the laboratory to analyze the heated vapors prior to the current tests. The eGo-CE6 coil temperature was previously reported by this laboratory [10], though the specific coil head tested in this work was unused.

**Table 1:** Sample descriptions and results for vaping cartridges and pods.

Brand (condition)	Type/yr obtained			SEM/EDS^a^		XRF	Tank walls
		Heating element type	Additional wicking/insulation & seals	Filament	Battery contact		
CCell #1 (used by EVALI patient)	THC cartridge/2019	Ceramic	Semi-synthetic fiber wrap with Si-rich rubber plug	Ni, Cr, Fe (wire leads: Ni; wire end: Ni, Fe, Cr, (Co), S)	Ni, Cu, Zn; (Si-rich rubber gasket)	Filament: Ni, Cr, Fe; Battery contact end cap: Ni, Cu, Zn, Pb, Fe	plastic
TKO (used by EVALI patient)	THC cartridge / 2019	Ceramic	Semi-synthetic fiber wrap with Si-rich rubber plug	Ni, Cr, Fe (wire leads: Ni)	Ni, Cu, Zn, Sn	(not tested)	glass
CCell #2 (unused/empty)	THC cartridge/2019	Ceramic	Semi-synthetic fiber wrap with fiberglass plug	Ni, Cr (wire leads: Ni)	Ni (Si-rich rubber gasket)	(not tested)	plastic
Hermes (unused/empty)	THC cartridge/2019	Ceramic	Si-rich rubber envelope and metal insert	Ni, Cr, Fe (wire leads: Ni; insert: Fe, Cr, Ni; sheath: C, F)	Ni (Si-rich rubber gasket)	(not tested)	glass
Unknown #1 (unused/filled)	THC cartridge/2018	Fiberglass wick	Si-rich rubber cap w/embedded fiberglass	Ni, Cr (wire leads: Ni)	Ni, Zn, Sn (Co) (Si-rich rubber gasket)	Filament: Ni, Cr; Battery contact end cap: Ni, Cu, Zn, Pb, Fe (Co)	plastic
Unknown #2 (unused/filled)	THC cartridge/2018	Dual fiberglass wick	Woven fiberglass wrap	Ni, Cr (wire leads: Ni; sheath: C, F)	Ni, Cu, Zn, Sn (Co) (Si-rich rubber gasket)	(not tested)	glass
JUUL (used in the lab)	Nicotine pod /2019	Fiberglass wick	none	Ni, Cr, Fe	Ni, Fe, Au	Filament + battery contact: Ni, Fe, Cr, Au	plastic
EGO-CE6 coil head (unused)	Nicotine device/2016	Fiberglass wick	none	Ni, Cr, Fe (wire leads: Ni; joint: Ni, Cr; sheath: C, F)	Ni	Filament: Ni, Cr, Fe, Zr; Coil head body: Ni, Cu, Zn, Pb, Sn, Fe	glass

^*a*^SEM/EDS types: ceramic = Si, O, Na, Al, P, K; fiberglass = Si, Al, O; semi-synthetic fiber = C with striated morphology; Si-rich rubber = Si, O, C [Ca, Ti, Zn].

The origins and operating voltages of the four THC cartridges from investigations are unknown, and their operating temperatures could not be tested due to the previous extraction of their liquids. The four new THC and nicotine products that were tested for comparison purposes had manufacturer-recommended power sources with batteries and operating voltages ranging from 200–1100 mAh and 2.8–6.0 V. It should be noted that various brands and types of power sources are available on the market, and a user may choose a different power source other than those recommended by the manufacturer. None of the manufacturers provided publicly available, validated measurement data for their internal heating temperatures.

All pods and cartridges were disassembled with the aid of a vise, pliers, hand saw, and tweezers to determine their common functional components and were documented with reflected-light stereozoom microscopes at 6.3x – 80x magnification (S8APO and S6D, Leica, Wetzlar, Germany). The inferred plastic or glass construction of the tank walls for these devices is also listed in Table 1 based on their brittleness during disassembly. Except in one case, the tank walls were not analyzed further, as they were judged less likely to be exposed to direct heat.

Components from two of the THC cartridges and both nicotine devices were mounted on Mylar X-ray film and analyzed using a portable XRF (Innov-X Alpha-4000 SL, Woburn, USA) with bench testing stand. Energy calibration was verified with a 316 steel standard. The detection limit varied by element between 10 ppm—1%. The minimum detectable atomic number was 15 (P, phosphorus). The spatial resolution of the XRF beam was approximately 1cm, and was convenient for integrating over entire pieces of the cartridge, such as the end caps containing the battery contacts.

Internal components from all eight pods and cartridges were analyzed by SEM/EDS using an FEI XL30 Environmental SEM with Noran System 7 EDS (Thermo Fisher Scientific, Madison, USA) and SEMView 8000 electronics (SEMTech Solutions, North Billerica, USA). Cartridge and pod components were further disassembled after portable XRF analysis to enable SEM-EDS imaging with 0.1 um spatial resolution. Samples were prepared on standard, aluminum SEM stubs with double-sided, carbon adhesive tabs and no conductive coatings applied. Samples were imaged at 20 kV and 20–2,500x using a tungsten source and an atomic-number-sensitive, back-scattered electron detector for rapid screening of heavy metals. X-ray energy-based element identifications were verified with a copper standard and had a spatial resolution of approximately 5 um. EDS quantitation is generally accurate only for flat specimens. Because some of these parts possessed substantial, variable topography on the order of 1 cm tall, elemental compositions were reported only in terms of qualitative presence/absence of major constituents, with an approximate 1% weight percent reporting limit.

Limited organic compound speciation was performed using a Nicolet iN-10MX FTIR Microscope with Omnic Picta software and a slide-on ATR detector with germanium crystal and MCT-A detector (Thermo Fisher Scientific, Madison, USA). Spectra were acquired with 4–8 cm^-1^ spectral resolution from 700–4,000 cm^-1^. Identification of component mixtures was assigned manually with the aid of quantitative FTIR spectral searches and in-house and commercial libraries of over 100,000 spectra. The estimated limit of detection is on the order of 1%.

## Results and discussion

### Cartridge and pod components

**Fig 1:**
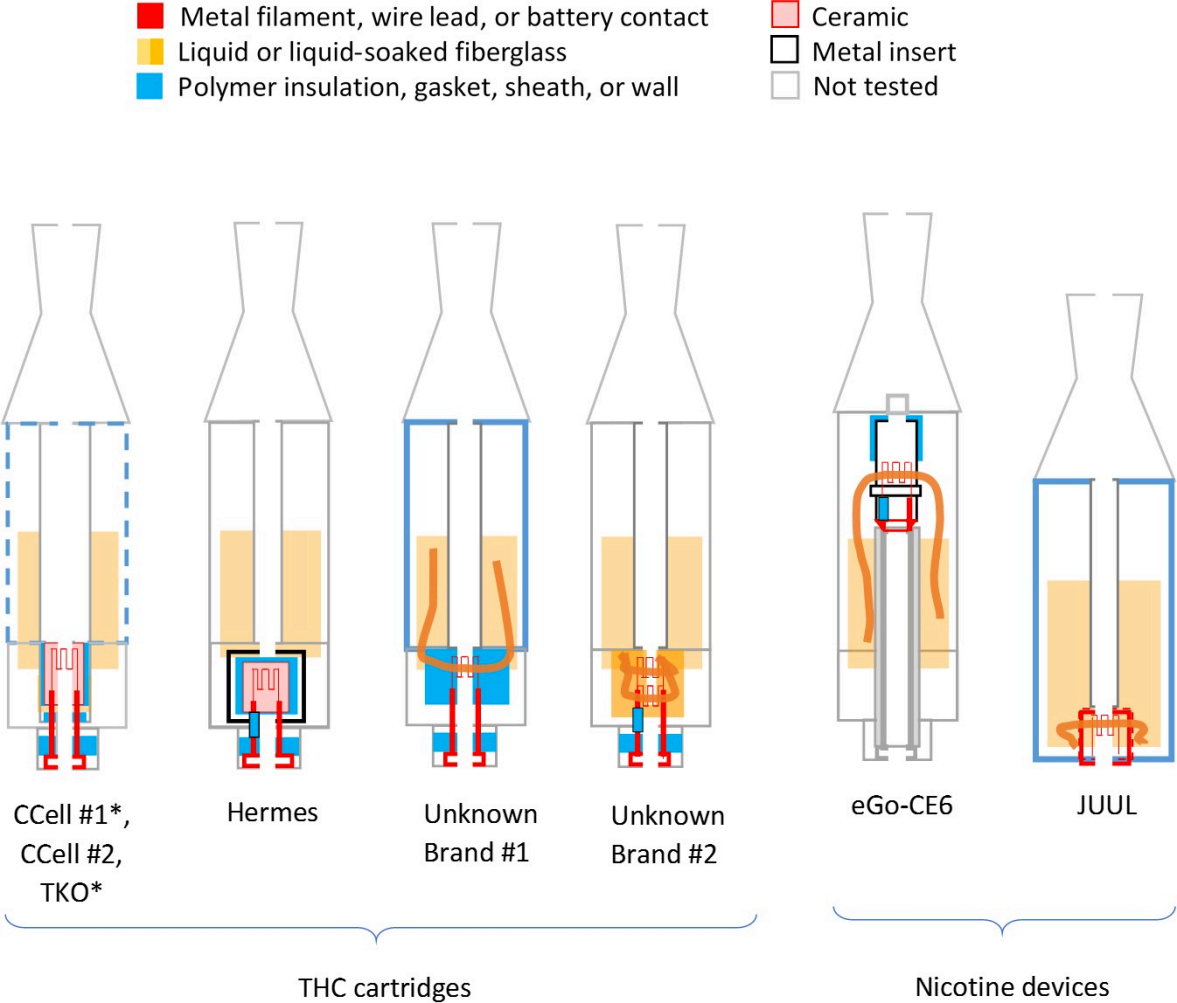
Schematic of THC and nicotine devices and functional elements revealed via disassembly. The EVALI patient cartridges are denoted with a (*). Note the fluorinated, polymer wire sheaths observed inside the Hermes, Unknown #2, and eGo-CE6 designs. Two minor variations were observed between brands in the left-most cartridge type: 1) the plug below the ceramic in one of the tested CCell cartridges was made of fiberglass, not a polymer. 2) the TKO tank walls were made of glass, not polymer.

Typical pod and cartridge components revealed during disassembly are summarized in Fig 1. In most types, a 4mm diameter, circular battery contact with 5mm diameter, polymer gasket was located at the bottom of each cartridge or pod. Above it, each heating element consisted of a filament, either wrapped around a fiberglass wick or embedded in a ceramic, adjacent to holes that admit liquid from the surrounding tank. Some designs also added a polymeric or fiberglass wicking/insulation wrap or plugs. Most filaments were joined to two long wire leads, which sometimes possessed a protective polymer sheath around one of the brazed connection points. These designs allow for vape liquid to be drawn in and vaporized by the heating element, then inhaled up through the air tube. The hot filaments, wicks, and insulation are in direct contact with both the liquid and inhaled airstream. It is unclear to what extent the wire leads or battery contact are similarly exposed. Many of these design features are consistent with later-generation e-cigarette designs reported by Williams and Talbot [9].

**Fig 2:**
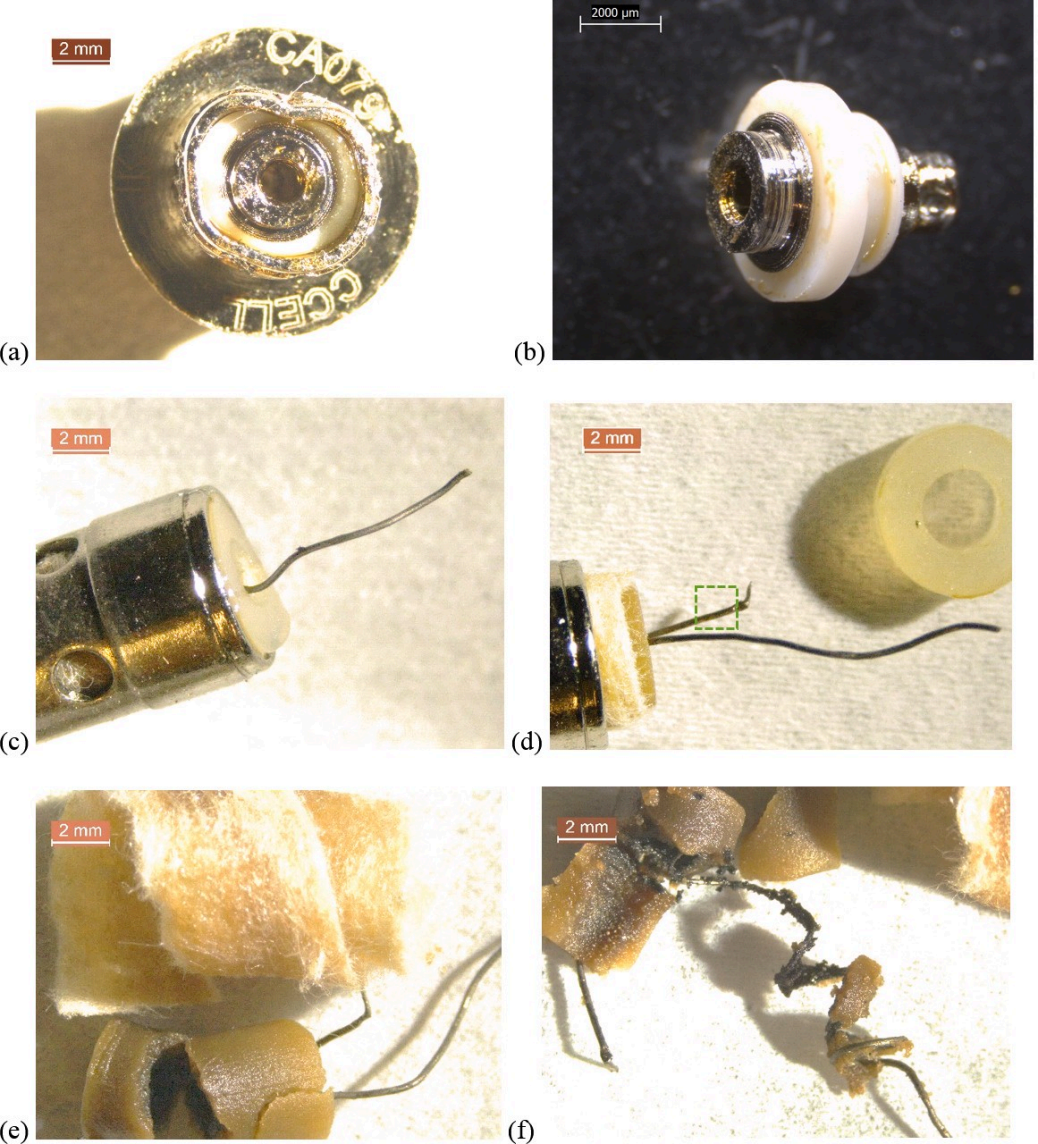
6.3-10x stereozoom microscope images of components from used CCell cartridge associated with EVALI case. a) bottom of cartridge end cap showing battery contact. b) battery contact in white polymer gasket. c) air tube with fluid holes and filament wire protruding through rubbery end cap. d) filament wire leads, ceramic, and fibrous insulation partially exposed. See Fig 11D for composition of boxed region. e) ceramic material broken to liberate filament. f) black, burnt material inside oil-soaked ceramic, suggesting exposure to high temperatures.

**Fig 3:**
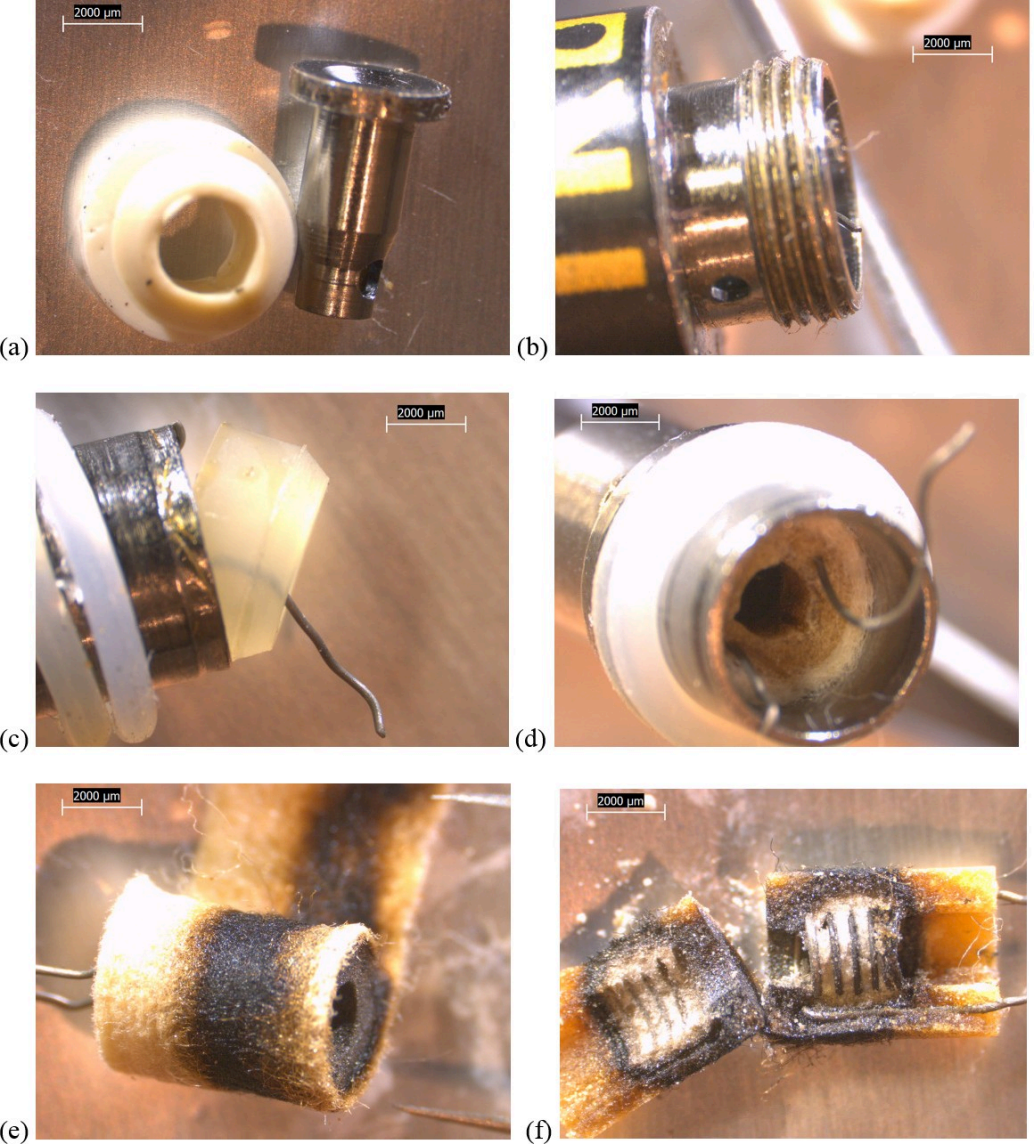
10x stereozoom microscope images of components from used TKO cartridge associated with EVALI case. a) battery contact and white polymer gasket b) filament wire leads exposed when battery contact removed c) polymer plug at end of tube. d) filament wire leads embedded in cylindrical ceramic, itself wrapped in fibrous wicking/insulation. e) charred material suggesting high temperatures on fibrous insulation and downstream end of ceramic. f) inside of oil-soaked ceramic showing embedded filament coils and no burn marks where there is no oil.

**Fig 4:**
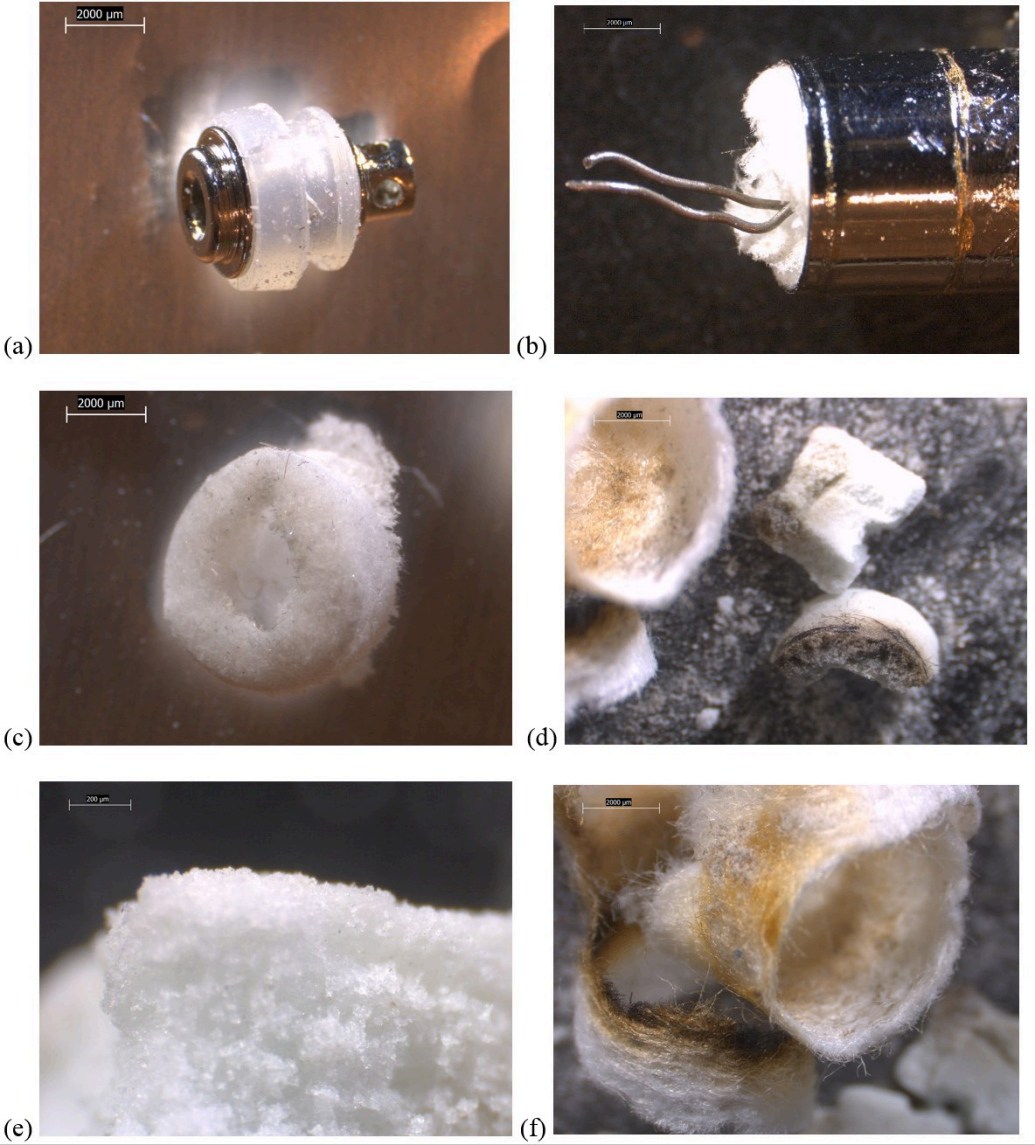
10-80x stereozoom microscope images of components from new CCell THC cartridge. a) battery contact in clear, rubbery gasket, of a slightly different design than that shown for the CCell and TKO cartridges. b) wire leads to filament embedded in fibrous insulation plug a) fibrous plug detail, made of a different material than the CCell and TKO cartridge end plugs. b) broken apart ceramic and fibrous wicking/insulation showing oil-free white color, and burn marks that are less severe than on the used CCell and TKO ceramics. C) detail of porous ceramic. D) detail of insulation.

Some design differences between device types were evident. All of the newer THC cartridges possessed ceramic heating elements. The filaments in the TKO and both CCell cartridges were embedded in 6 x 4 mm, tube-shaped ceramics (Figs 2–4). The fibrous wicking/insulation wrap in this type appeared to completely fill the cylindrical volume of each heating element. The CCell and TKO cartridges belonging to EVALI patients exhibited extensive charred, blackened material on the inside and outside of the oil-soaked ceramics and insulation, suggesting burned THC liquid and very high temperatures at the filaments (Figs 2E and 2F and 3E and 3F). Careful dissection of the TKO ceramic revealed that only the areas of the ceramic soaked with oil were blackened (Fig 3F). The unused CCell cartridge (#2) also showed minor burn marks, perhaps as the result of manufacturer testing (Fig 4D and 4F). Note that the new CCell was manufactured with slightly different materials than the used CCell, including a clear battery contact gasket instead of white, different heating element end plug (fibrous instead of polymer), and different mouthpiece/drip tube design (not shown here).

**Fig 5:**
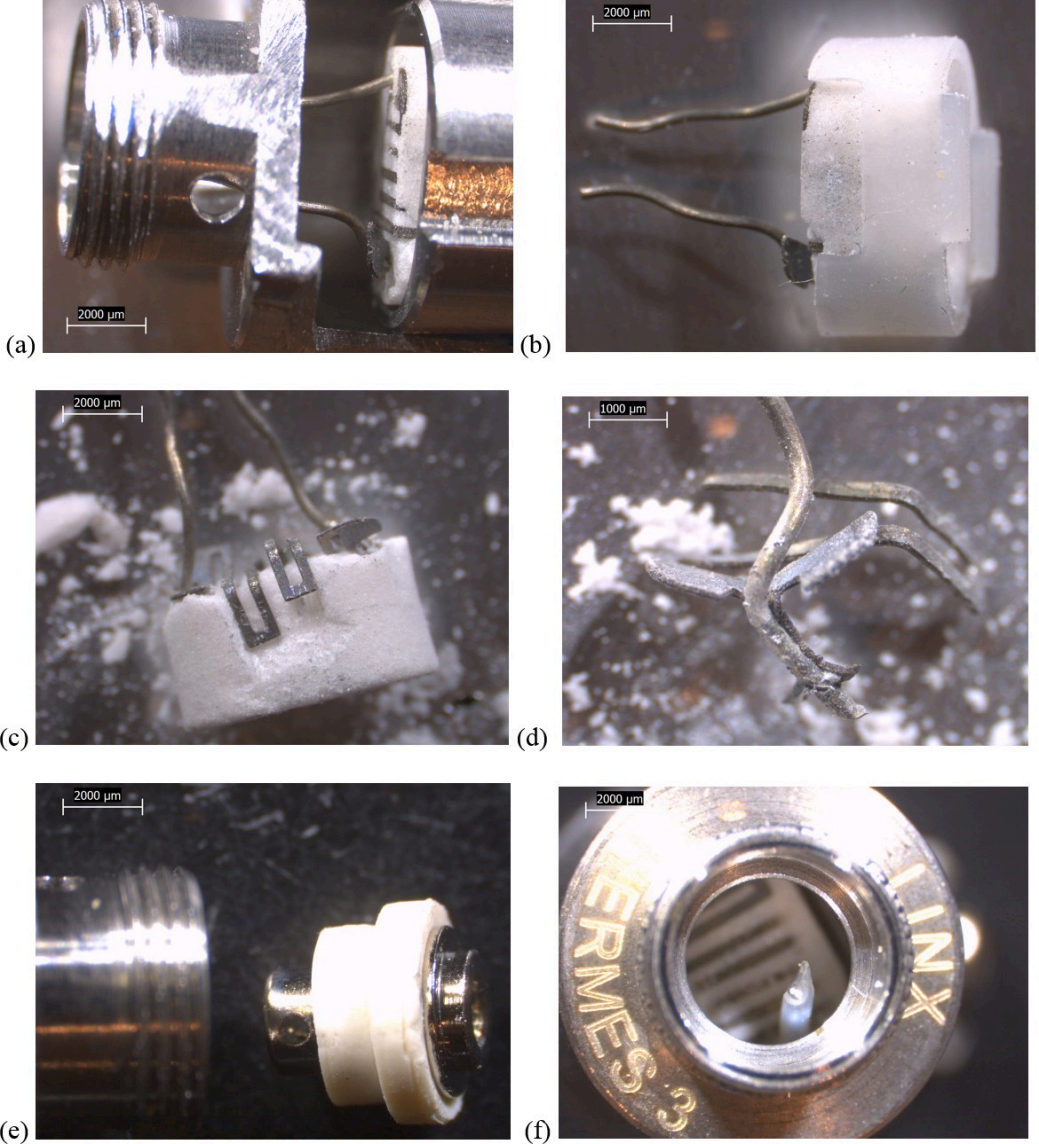
10-20x stereozoom microscope images of components from new Hermes THC cartridge. a) battery contact removed and metal end cap wall sawed off, showing filament embedded in ceramic, housed in a clear rubbery envelope inside a metal insert. b) heating element removed, showing rubbery envelope. c) ceramic chipped away to reveal square geometry of filament turns. d) filament removed from ceramic, showing brazed wire lead. e) battery contact with white, rubbery gasket. f) view towards heating element with battery contact removed, showing clear polymer sheath around one wire lead.

The heating element from an unused Hermes THC cartridge (Fig 5) was a 6 x 3 x 2 mm ceramic in the form of a solid block, different than the tube designs in Figs 2–4. The filament was also of a different design, with a rectangular cross section and a rectangular-planar arrangement, rather than cylindrical coils. The ceramic was housed inside a 7 x 3 x 3 mm, clear rubbery envelope, encased in a metal insert. The end of one wire lead was wrapped in a clear, cylindrical sheath measuring approximately 1.5 x 0.5 mm (Fig 5F). The sheath may have been used to prevent the wire lead from shorting out.

**Fig 6:**
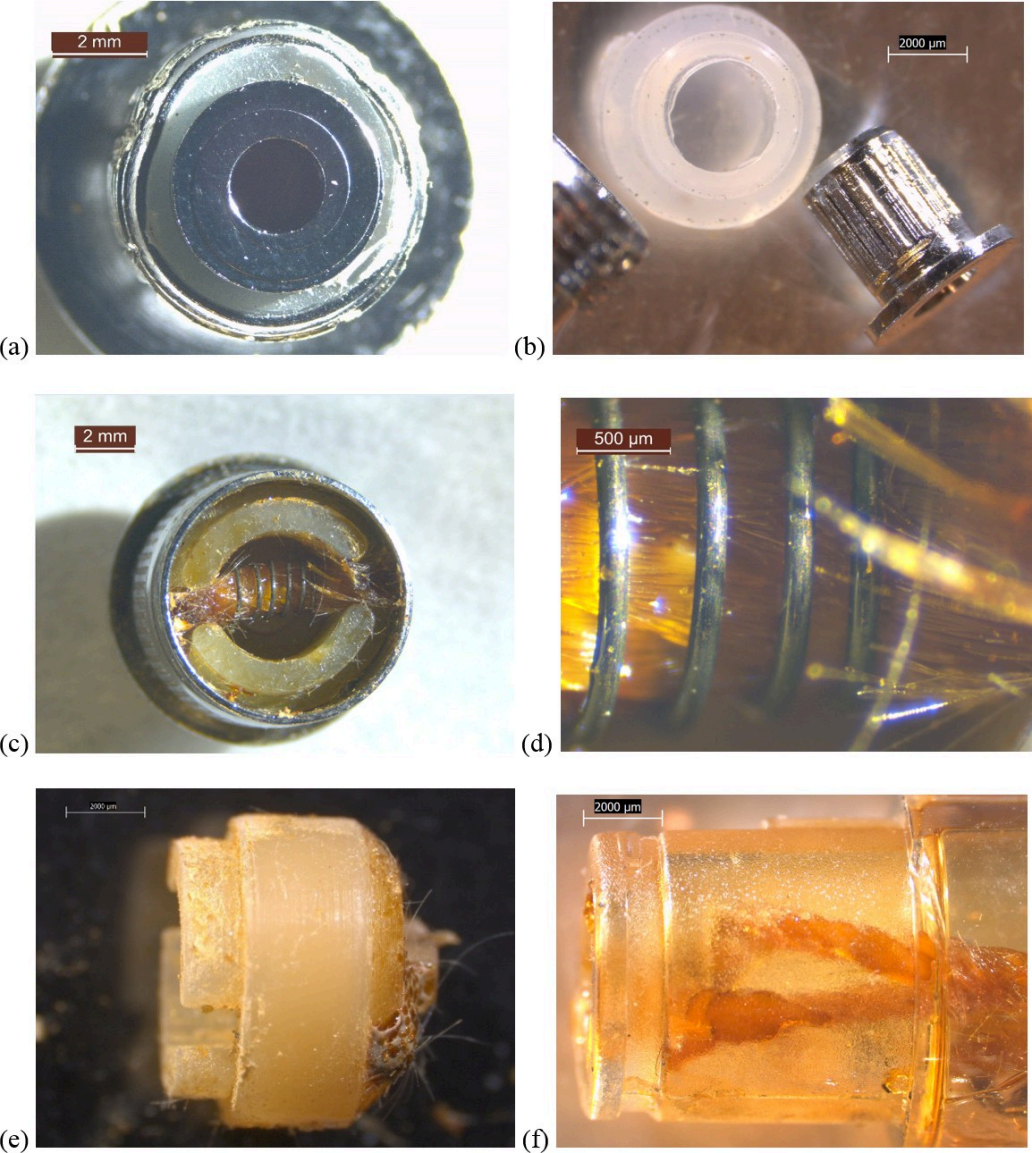
6.3-40x stereozoom microscope images of components from new THC cartridge “unknown #1”. a) upstream end of cartridge with battery contact b) battery contact and clear polymer gasket. c) downstream end of metal cartridge end cap showing fibrous wick inside filament, itself embedded in rubbery cap. d) same as (c) at higher magnification. e) side view of rubbery cap with brown substance and fibers where filament wire leads passed through. f) plastic tank walls and wick pieces.

**Fig 7:**
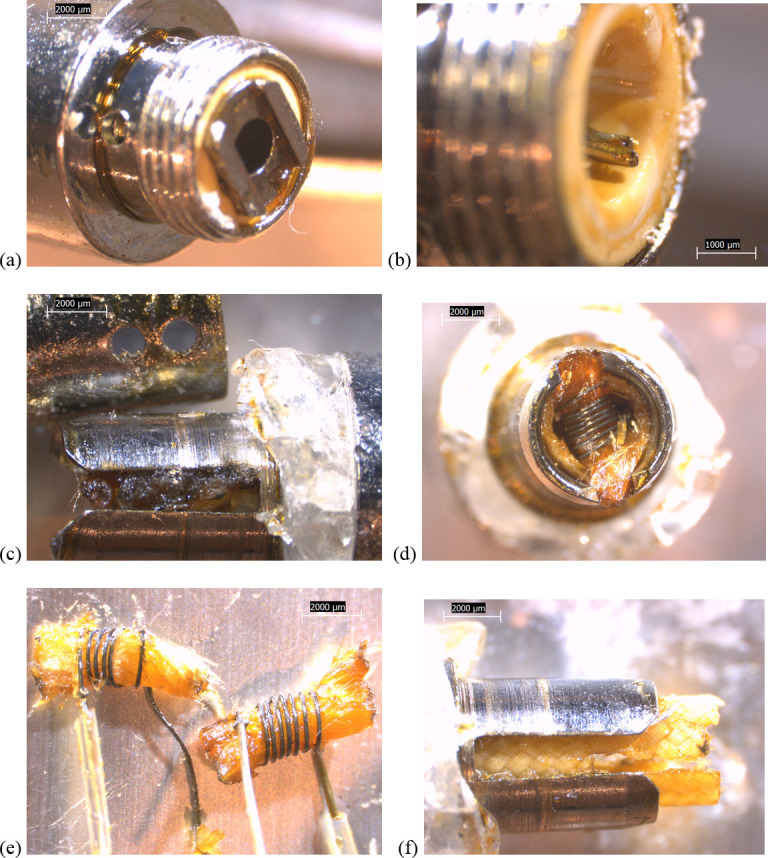
10x stereozoom microscope images of components from new THC cartridge “unknown #2”. a) battery contact with rectangular rails, inside white polymer gasket. b) wire leads visible with battery contact removed. c) side view of air tube with two wick access holes, and filament housing slot showing two stacked filaments obscured by wick material, insulation, and oil residues. Broken tank glass is also visible. d) bottom view of bottom-most filament and wick. e) both filaments with wicks and one polymer sheath each. f) quilted fibrous insulation with slot cut out to expose filaments to oil.

In contrast, the heating elements in the two unused THC cartridges of the older, unknown brands (Figs 6 and 7) possessed non-ceramic filaments wrapped around wicks, with simpler wicking/ insulation that did not completely fill the surrounding spaces. Unknown cartridge #2 possessed two filaments, each with a clear sheath around one of the wire lead joints (Fig 7E).

**Fig 8:**
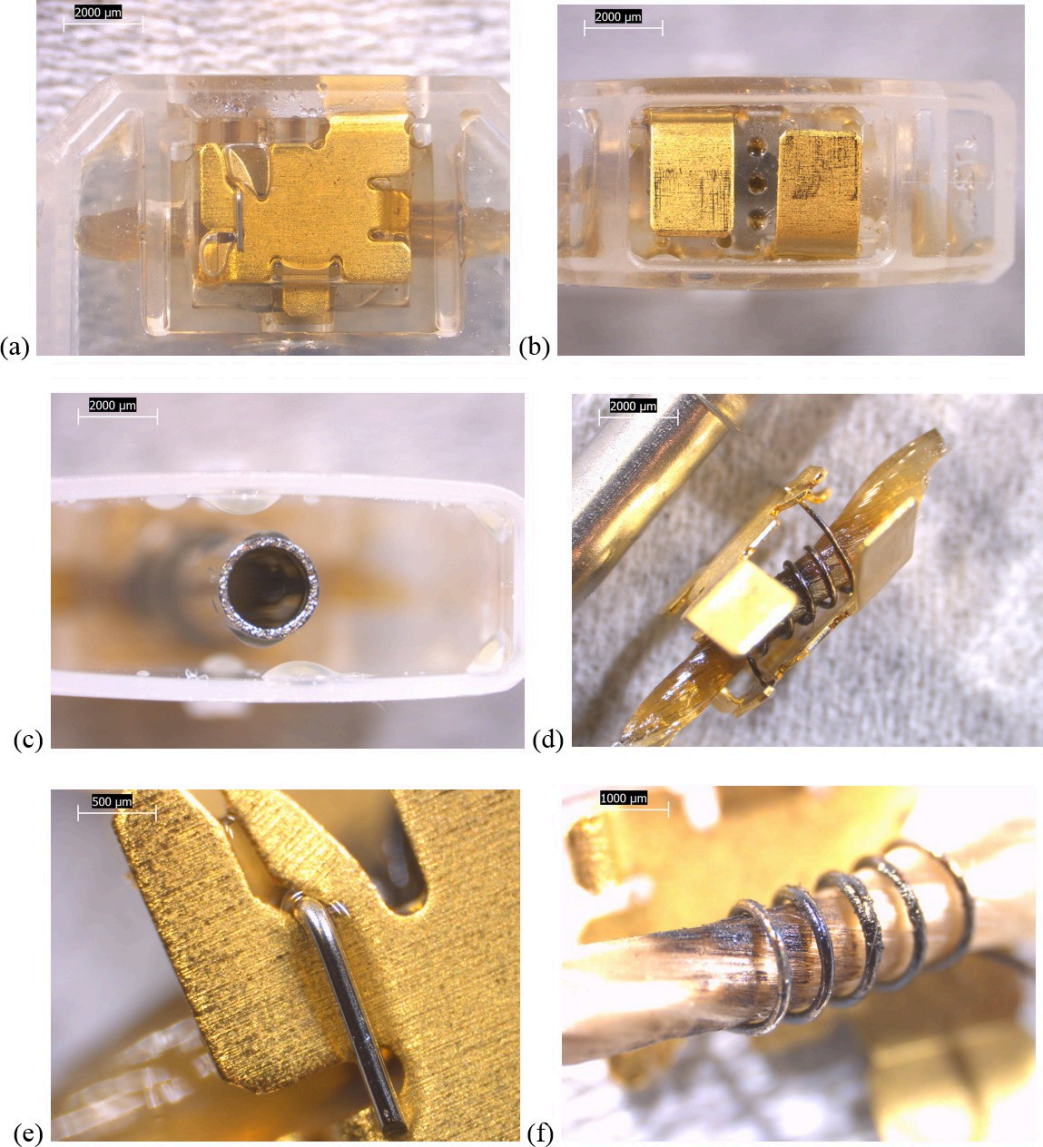
6.3-10x stereozoom microscope images of components from used JUUL pod. a) side view of battery contact–filament -wick assembly inside clear plastic pod. b) top view of (a) showing battery contact tabs. c) bottom view of pod showing metal air tube. d) same view as (b) but removed from pod, showing darkened filament with oil-saturated wick. e) non-brazed connection between filament and battery contact. f) top view of filament and evaporated wick, showing partially burned surfaces.

The used JUUL nicotine pod (Fig 8) differed from the THC cartridges in several respects: 1) there was no wicking/insulation wrap or wire leads, 2) the much larger battery contact plates were integrated into the same space as the filament and wick, and 3) all were located within plastic dividers integrated into the rectangular plastic tank walls. Localized regions of the used JUUL filament and wick surfaces were partially blackened, presumably due to high temperatures, though the amount of burnt material was substantially less than the THC cartridges used by EVALI patients (Figs 2 and 3).

**Fig 9:**
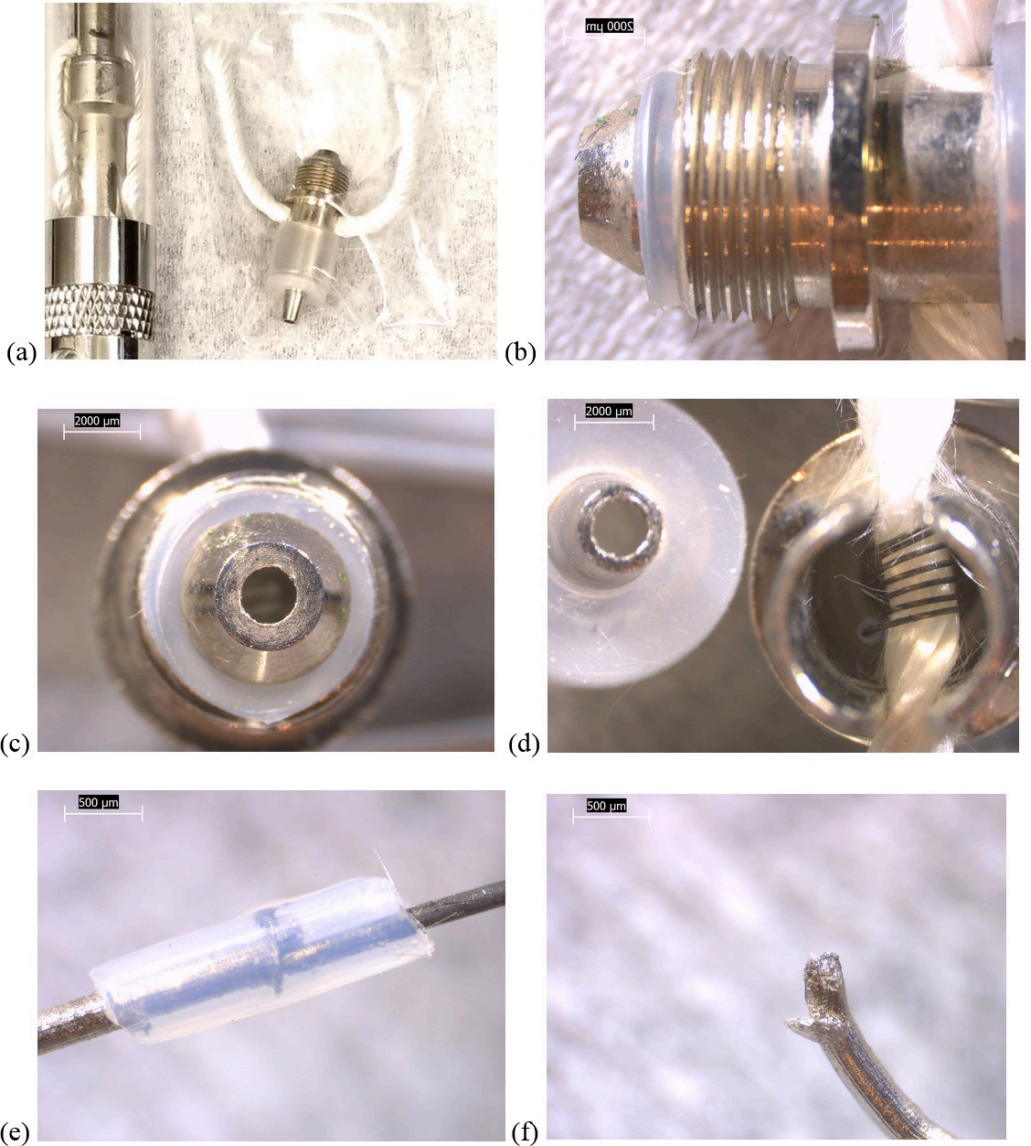
Images of components from new EGO-CE5 coil head. a) camera image showing coil head with wick, next to unit containing empty tank, air tube, and battery contact. b)–f) 6.3-40x stereozoom microscope images: b) side view of threaded battery contact end. c) top view of (b). d) opposite view of coil head with air tube nozzle removed and slotted wick-filament holder visible. e) Clear polymer sheath wrapped around one filament-wire joint. f) End of wire lead that touches battery contact.

The EGO-CE6 coil head for a nicotine device (Fig 9) also appeared to possess no insulation wrap. In addition, its battery contact-facing end connects to the battery via a long metal cylinder around the air tube rather than directly (Fig 1), and the coil head is closer to the mouthpiece than any of the other designs. Otherwise, it possessed some similar features to the THC cartridges, including a metal cylindrical barrel shape and brazed wire leads, one with a clear sheath (Fig 9E).

### Metal, ceramic, and polymer elemental compositions

**Fig 10:**
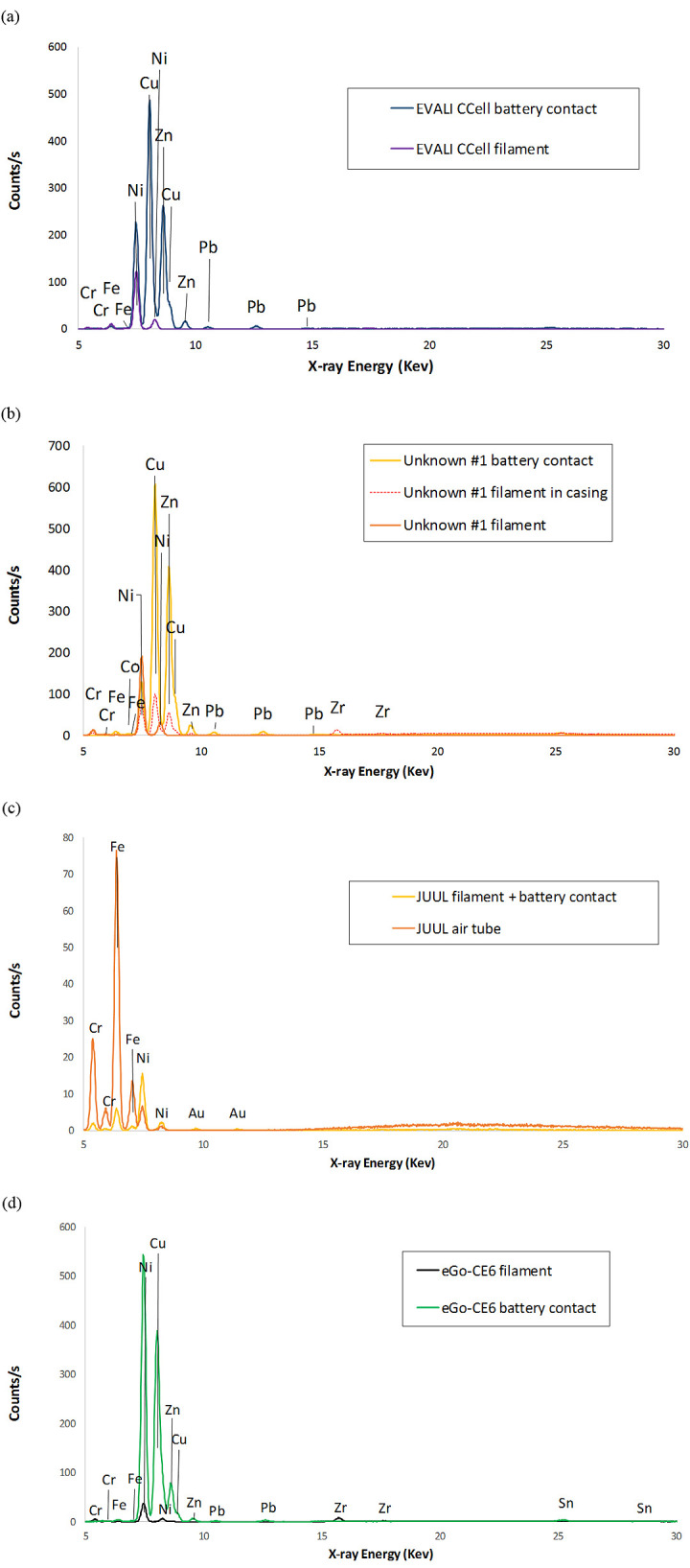
Portable XRF results. a) EVALI patient CCell cartridge b) new THC cartridge Unknown #1. c) used JUUL pod d) new EGO-CE5 coil head. The tested battery contacts for (a) and (b) included the entire end caps, were integrated with the filament for (c), and comprised the entire bottom end of the coil head body for (d).

Portable XRF analyses performed on 11 components of the four devices are shown in Fig 10 and Table 1. The filaments in both tested THC cartridges yielded primarily Ni, and the battery contacts encased in their respective cartridge end caps exhibited copper (Cu), Ni, and zinc (Zn), with smaller Pb, Cr, iron (Fe), and zirconium (Zr) peaks. A minor Co peak was also present in the Unknown #1 cartridge. All minor Co identifications in this work obtained with XRF and SEM-EDS were considered inconclusive due to coincidence with a Si escape peak for Zn. XRF analyses were also performed on the rubbery cap and fibrous insulation from the EVALI-associated CCell cartridge, but no peaks above background were observed.

Portable XRF analyses of both nicotine device filaments (Fig 10C and 10D) yielded primarily Ni and Cr, plus minor Fe and Zr in the EGO-CE6. For the JUUL, XRF was performed on the filament, wick, and battery contact as one assembly, which also exhibited Fe and gold (Au). The battery contact for the EGO-CE6 was integrated into the end of the coil head body, and exhibited Cu, Ni, and Zn, with smaller Pb, tin (Sn), and Fe peaks. The JUUL air tube exhibited stainless steel elements Fe, Cr, and Ni. With the exception of Au, these elements in the nicotine devices were generally consistent with those in the THC cartridges.

**Fig 11:**
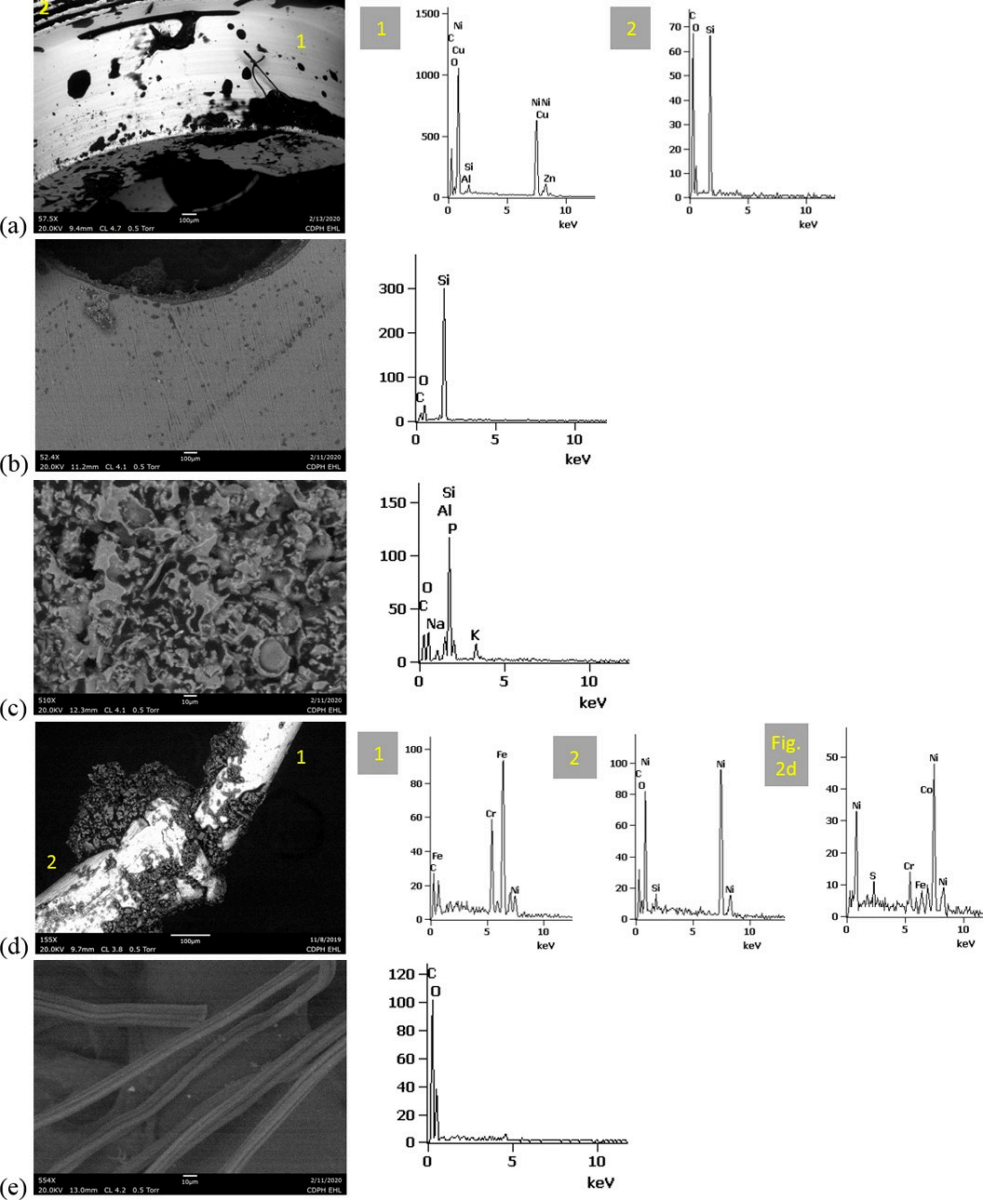
SEM-EDS for CCell cartridge associated with EVALI case. a) battery contact exhibiting Ni with Cu and Zn. Gasket exhibited Si with O and C. b) Polymer plug exhibiting Si with minor O and C. c) Ceramic exhibiting Si with minor O, Al, K, Na, and P. d) Filament with Fe, Cr, and Ni, and wire lead with Ni. A section of the wire lead near the battery contact (see Fig 2D) also had minor Fe, Cr, and Co. e) fibrous insulation with C, O, and striated profile.

**Fig 12:**
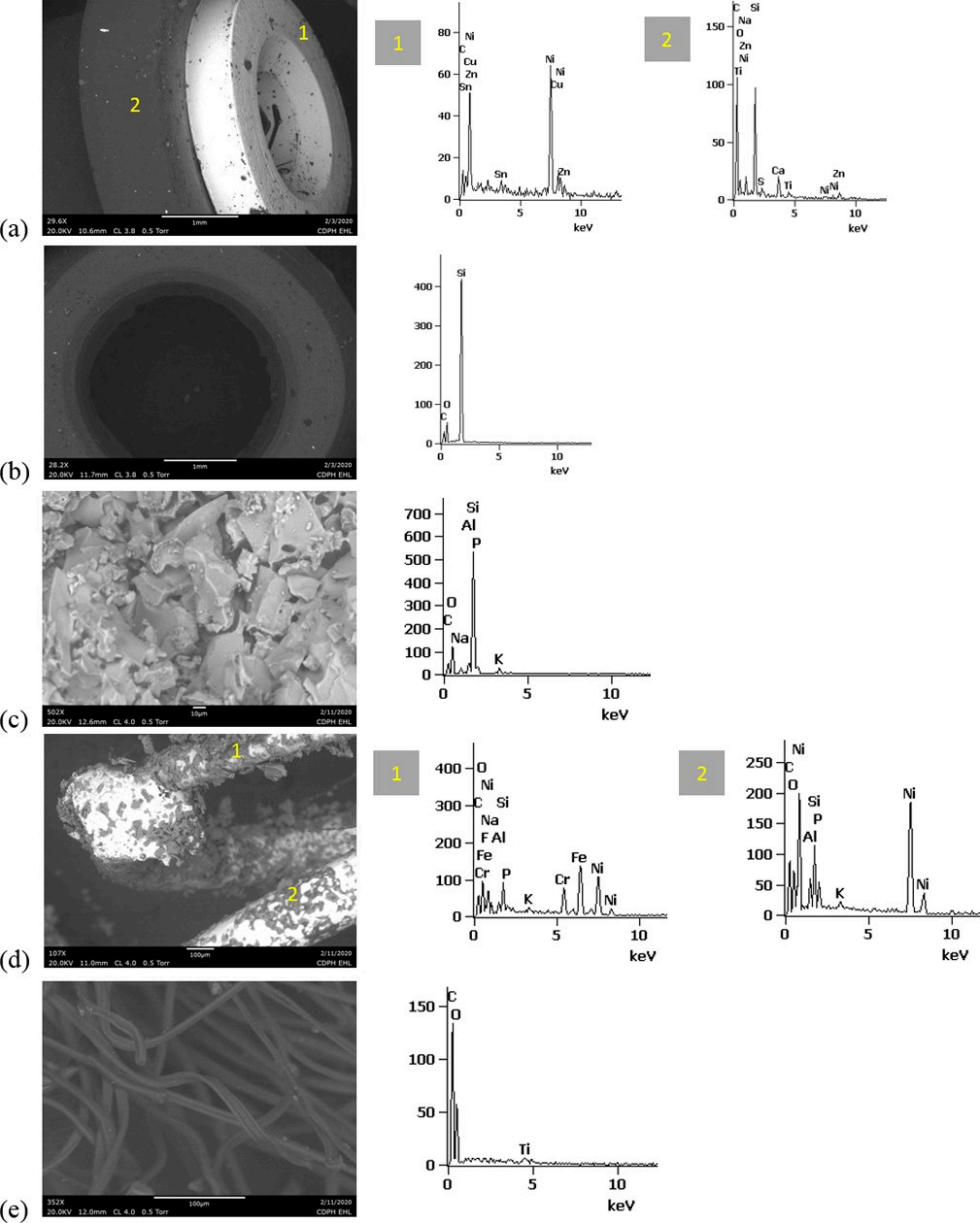
SEM-EDS for used TKO cartridge associated with EVALI case. a) battery contact exhibiting Ni with Sn, Cu, and Zn. Gasket exhibited Si with Ca, Ti, O and C. b) Polymer plug exhibiting Si with minor O and C. c) Ceramic exhibiting Si with minor O, Al, K, Na, and P. d) Filament Fe, Cr, and Ni, joined to wire lead with Ni. e) fibrous insulation with C, O, minor Ti, and striated morphology.

**Fig 13:**
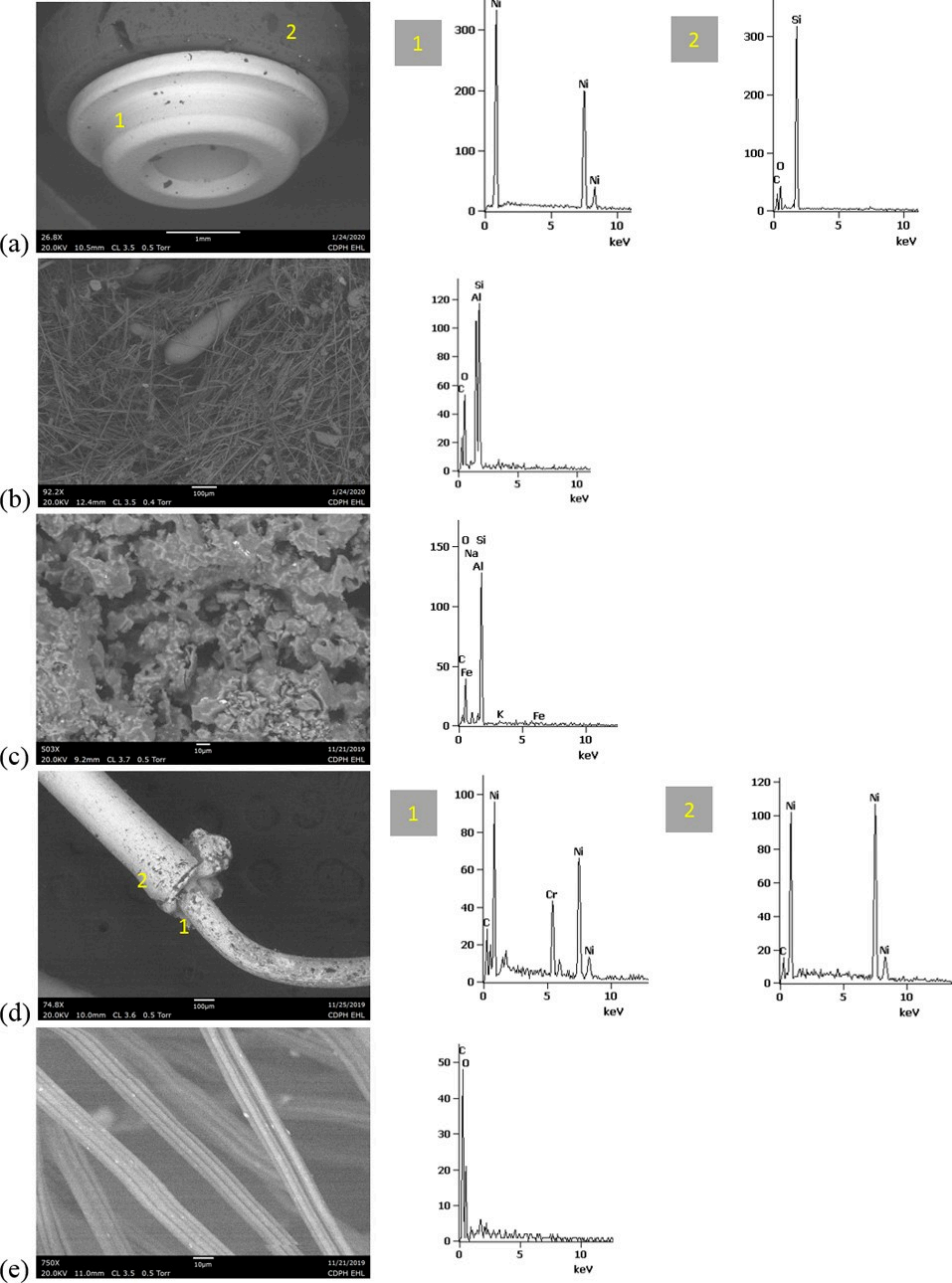
SEM-EDS for new CCell cartridge. a) battery contact exhibiting Ni and gasket exhibiting Si, C, and O b) fibrous plug with Si, Al, and O. c) ceramic with Si and minor Al, Na, Fe, and K. d) filament with Ni and Cr joined to thicker wire lead with Ni. e) insulation fibers with striated morphology exhibiting C and O.

**Fig 14:**
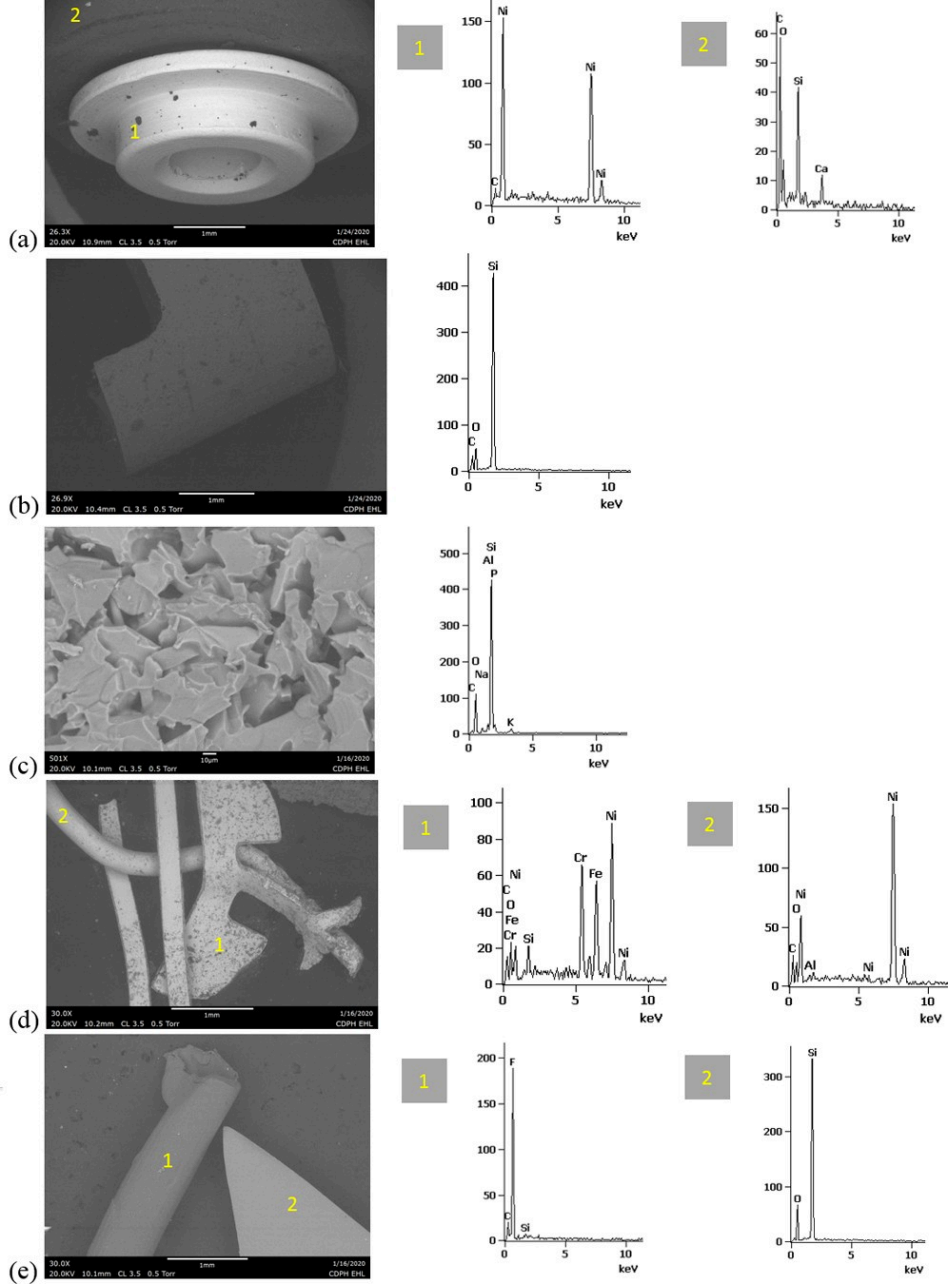
SEM-EDS for new Hermes cartridge components. a) battery contact with Ni and gasket with Si, C, Ca, and O. b) rubbery heating element envelope with Si and minor C and O. c) ceramic exhibiting Si with O, Na, P, and K. d) filament with Fe, Cr, and Ni and wire lead with Ni. e) wire sheath with primarily F and C and glass wall piece with Si and O.

**Fig 15:**
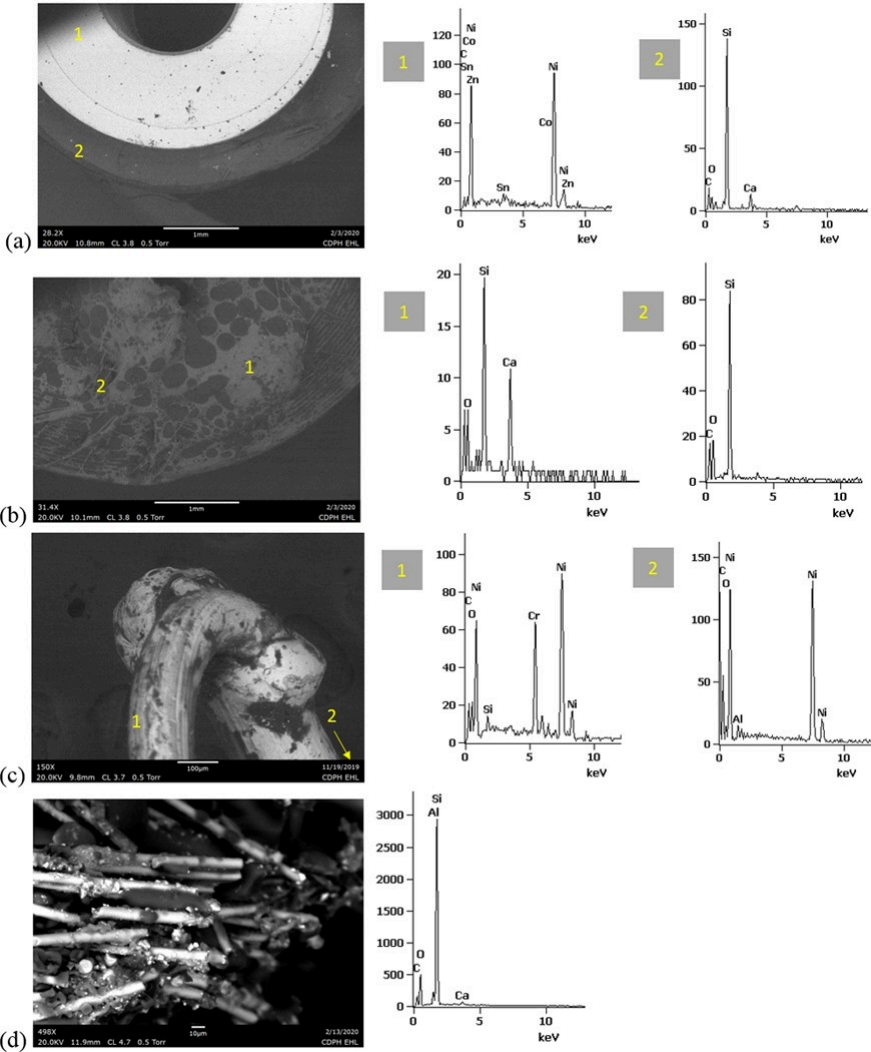
SEM-EDS for new THC cartridge “unknown #1”. a) battery contact exhibiting Ni with Sn, Zn, and minor Co, and gasket exhibiting Si, Ca, C, and O. b) rubbery plug with Si, Ca, and O and glass fibers exhibiting Si and O. c) filament with Ni and Cr joined to thicker wire lead with Ni (acquisition from beyond image frame). d) wick fibers exhibiting Si with minor Al, Ca, and O.

**Fig 16:**
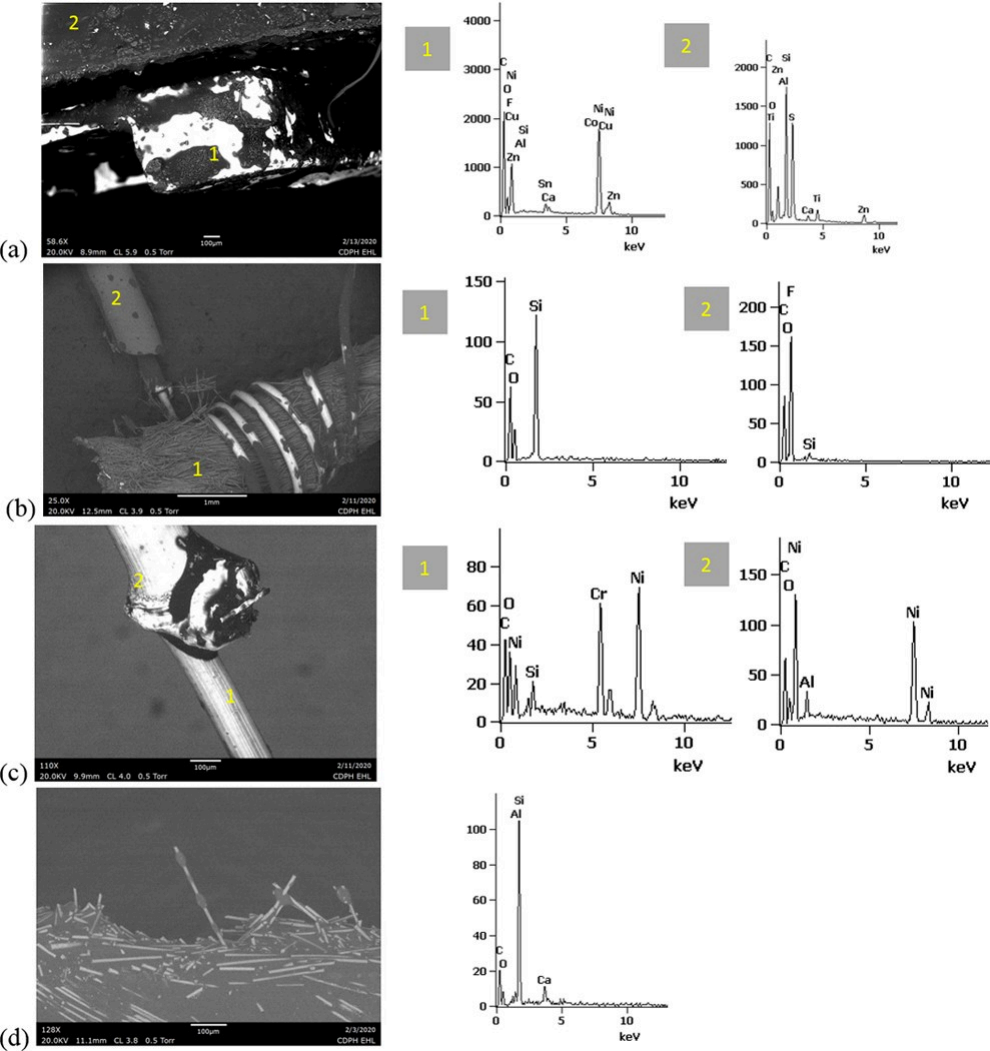
SEM-EDS for new THC cartridge designated “unknown #2”. a) battery contact exhibiting Ni, Zn, Sn, and minor Co and Cu, and gasket exhibiting Si, Ca, S, Ti, Zn, C, and O. b) wick exhibiting Si with O and polymer sheath exhibiting C and F. c) filament with Ni and Cr joined to thicker wire lead exhibiting Ni. d) insulation fibers exhibiting Si with minor Al, Ca, and O.

**Fig 17:**
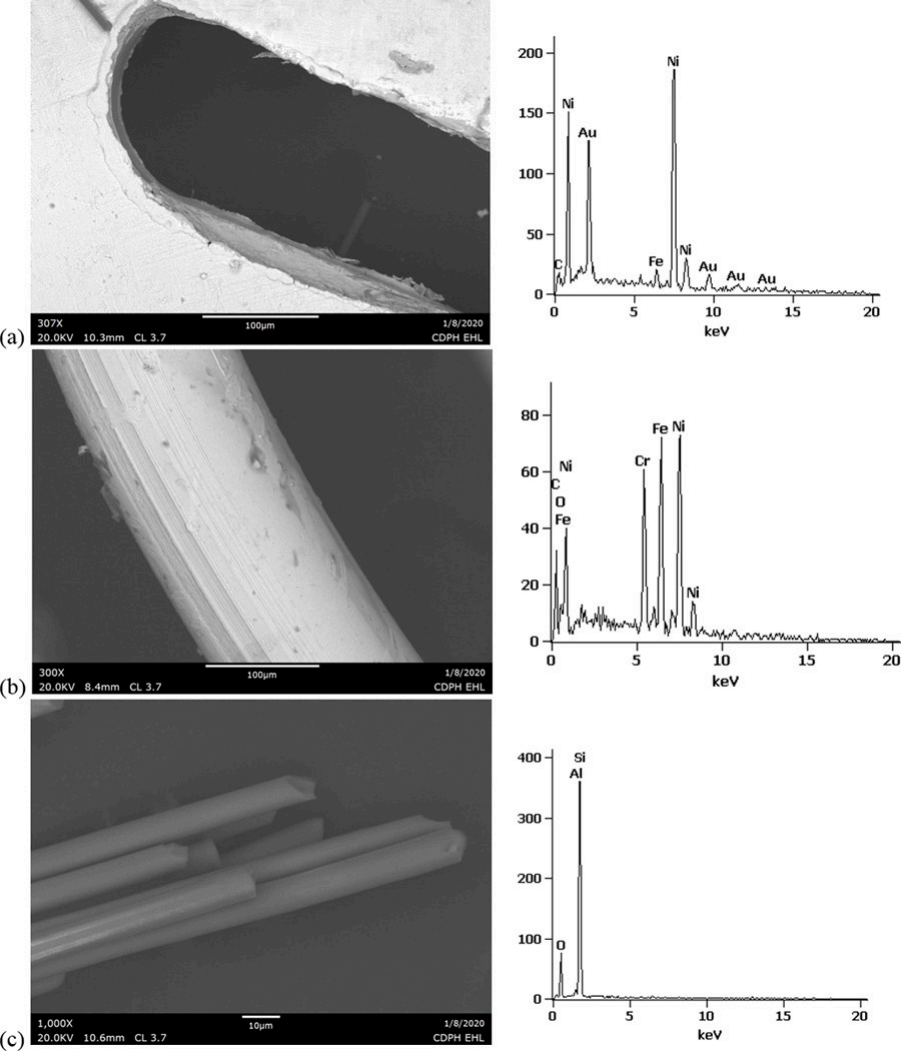
SEM-EDS for used JUUL components. a) battery contact showing brighter, Au enriched surface layer with Ni and Fe. b) filament with Fe, Cr, and Ni. c) glass fibers from wick, exhibiting primarily Si with minor Al and O.

**Fig 18:**
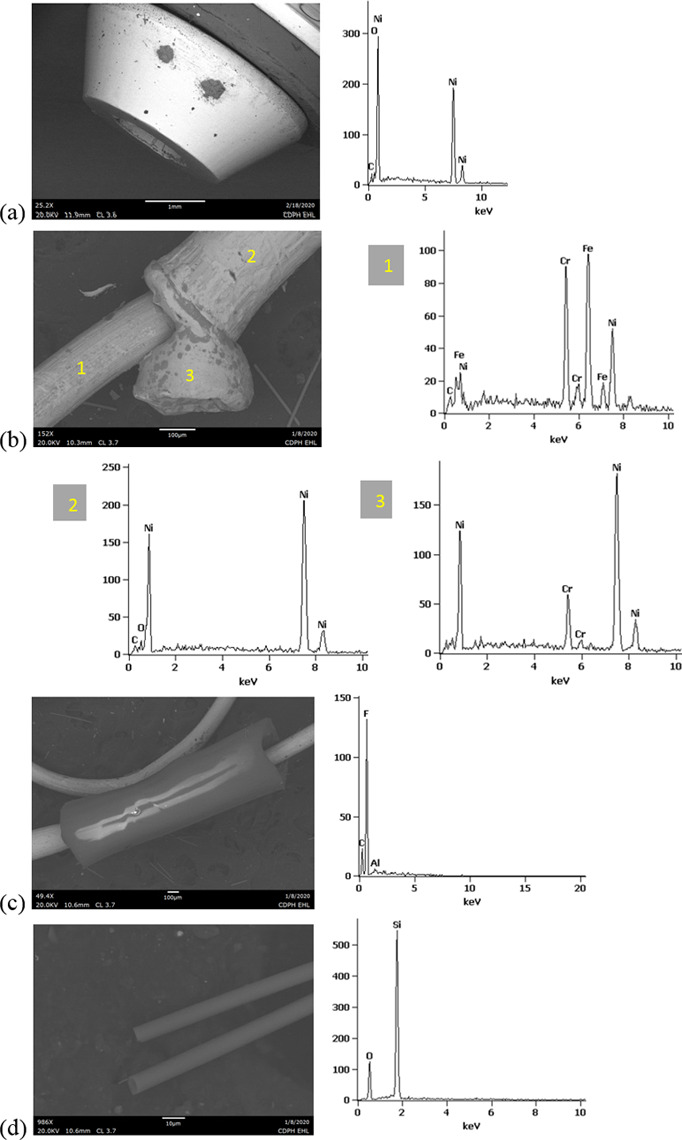
SEM-EDS for new eGo-CE5 components. a) Battery contact with Ni. b) Filament with Fe, Cr, and Ni, wire lead with Ni, and joint exhibiting Ni and Cr. c) sheath around filament-wire joint with primarily C and F. d) glass fibers from wick, exhibiting Si with minor O.

SEM-EDS results from 44 components, some of them obtained from further disassembly of pieces analyzed by XRF, are shown in Figs 11–18. The battery contacts from the THC cartridges were all very similar, composed primarily of Ni. Some of the battery contacts exhibited minor Cu, Zn, Sn, and inconclusive Co. All of the associated battery contact gaskets were composed of silicon (Si), oxygen (O), carbon (C), and sometimes Ca, Ti, or Zn, consistent with a synthetic or silicone rubber with inorganic fillers and pigments including silicates (hereafter referred to simply as “Si-rich rubber”). The JUUL’s uniquely designed battery contact tabs exhibited Ni and Fe with a surface layer enriched in Au (Fig 17).

The fibrous end plug in the unused CCell was fiberglass, primarily Si with minor Al (Fig 13), while the polymer plugs in the EVALI-associated CCell and TKO were both consistent with Si-rich rubber (Figs 11 and 12). The EVALI-associated CCell also had another gasket at the entrance to the mouthpiece consistent with Si-rich rubber (not shown in Figs).

The ceramics in the EVALI-associated CCell and TKO and the unused CCell and Hermes, were all dominated by Si, with varying amounts of aluminum, sodium (Na), potassium (K), and P. All had pore dimensions on the order of 10 um, presumably to allow oil to soak into the heating elements. The fibrous insulation wraps around these ceramics exhibited predominantly C and a striated morphology consistent with semi-synthetic rayon fibers (Figs 11E and 12E and 13E). The rubbery envelope around the Hermes ceramic exhibited the rubber elements Si, O, and C, and the metal insert was stainless steel with Fe, Cr, and Ni (not shown in Figs.) A piece of Hermes tank wall, presumed glass, was analyzed by SEM-EDS and was confirmed to exhibit Si and O.

All filaments were composed primarily of Fe, Cr, and Ni and were brazed to wire leads composed primarily of Ni, except for the unused CCell and two unknown brands, whose filaments were composed of Cr and Ni only. A wire lead from the EVALI-associated CCell had one region at the battery contact end that exhibited Ni, Fe, Cr, inconclusive Co, and sulfur. The JUUL did not possess wire leads. The eGo-CE6 was the only filament that possessed a slightly different metal signature at the joint, Cr and Ni, perhaps to aid in the bond (Fig 18). Because no additional metals such as Pb or Sn were detected at the filament-wire lead joints, the connections for all of the tested designs are likely brazed, rather than soldered as in older generation nicotine devices [22]. Individual fibrils in all tested wicks were composed of fiberglass, exhibiting primarily Si and O, sometimes with minor Al or Ca.

The polymer sheaths around one wire lead each in the Hermes, Unknown #2, and eGo-CE6 all exhibited C and a strong fluorine (F) peak, consistent with a fluorinated polymer such as FEP (fluorinated ethylene propylene) or PTFE (polytetrafluoroethylene), or possibly a polymer coated with PFAS (perfluorinated alkylated substances) or PFOS (perfluorooctanesulphonate) (Figs 14, 16 and 18).

**Fig 19:**
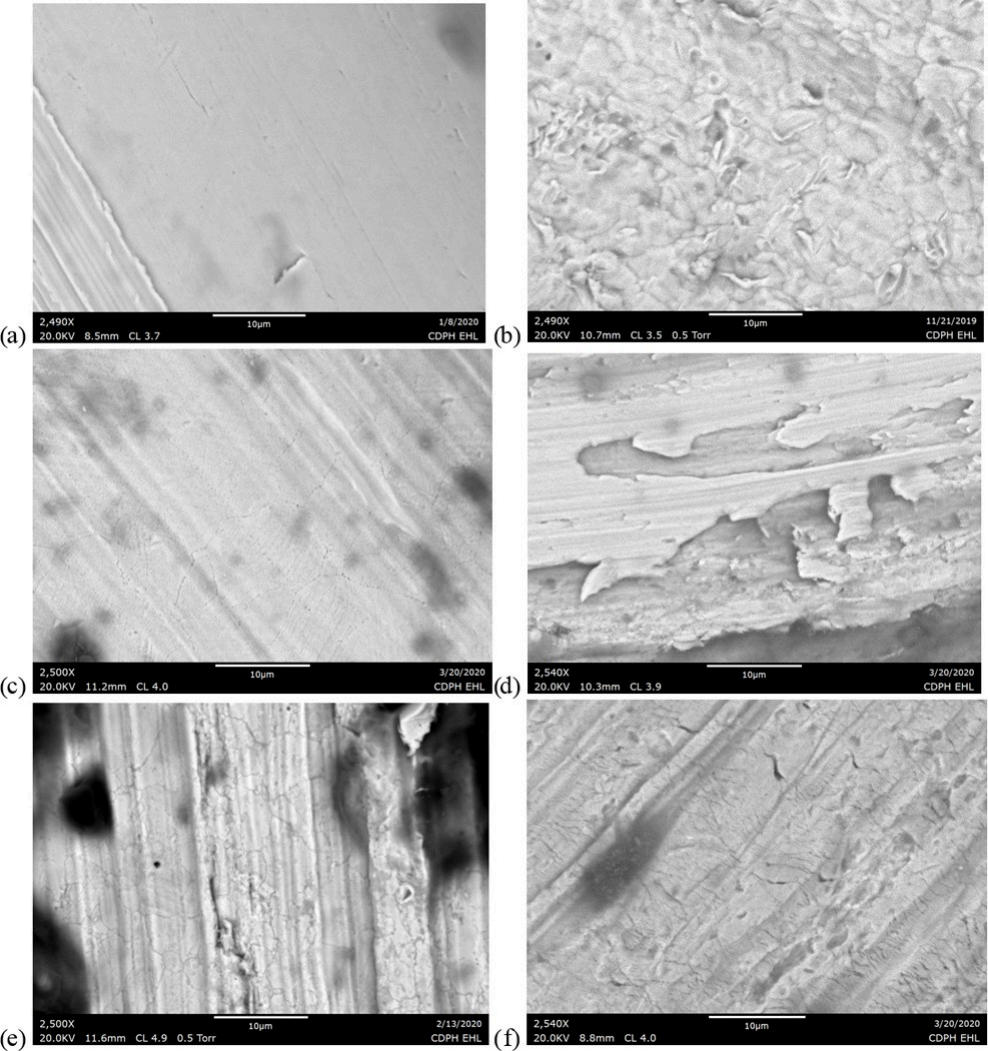
2,500x SEM images of metal surface conditions in new and used wires. a) used JUUL filament. b) new Cell wire lead. c) used CCell filament from EVALI patient d) used CCell wire lead. e) used TKO filament from EVALI patient. f) used TKO wire lead.

Higher magnification SEM images of the filaments and wire leads revealed the most surface damage in the used EVALI-associated cartridges. New filament and wire lead surfaces generally exhibited smooth surfaces with prominent grooves, similar to the used JUUL filament shown in Fig 19A. The new CCell filament uniquely possessed a mottled finish with carbonaceous surface regions, possibly representing a coating or surface finish created during manufacture (Fig 19B). In in the two used, EVALI-associated cartridges only, networks of microscopic cracks approximately 0.1 um wide were observed on the filament and wire lead surfaces (Fig 19C–19F). Some gross surface blemishes and deformations on the order of 10–100 um were observed on all filaments and wire leads, especially in the case of the JUUL where the filament was inserted and bent mechanically into notches in the battery contact rather than soldered or brazed. However, for the used EVALI wire leads, these sites appeared to be exceptionally prone to delamination and flaking (Fig 19D). Overall, the enhanced filament and wire lead degradation in the EVALI-associated cartridges is consistent with the higher degree of charring observed on and around those cartridges’ heating elements.

**Fig 20:**
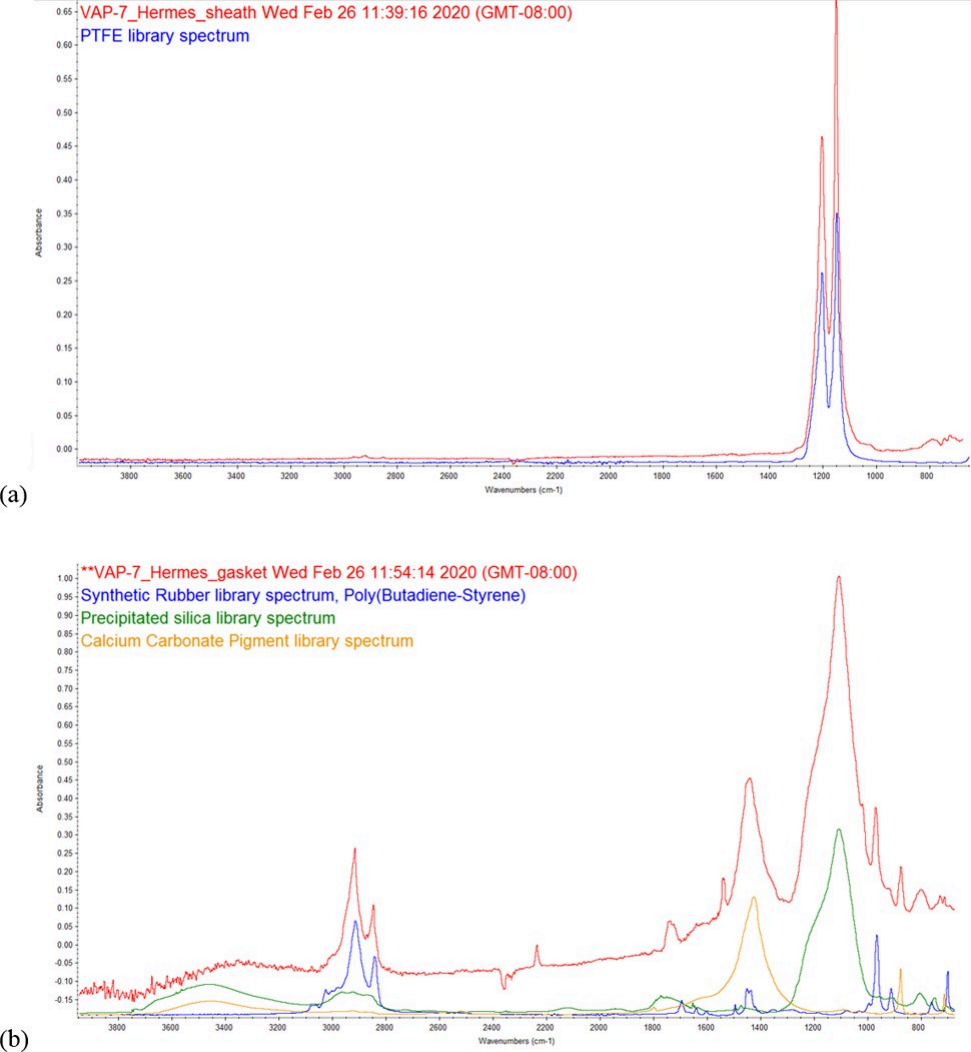
FTIR micro-ATR spectra for polymer components of Hermes cartridge. a) clear sheath from wire lead with library match to PTFE. b) battery contact gasket with matches to synthetic rubber and inorganic pigments (silica, calcium carbonate).

FTIR of two polymer components in the Hermes cartridge are shown in (Fig 20). The best match to the wire lead sheath was PTFE, and the best match to the battery contact gasket was synthetic butadiene-styrene rubber plus silica and calcium carbonate. These results are consistent with the SEM-EDS results for these items (Fig 14A–14E.) The library spectrum for Si-rich kaolin clay was similar to pure silica but is a less likely candidate due to the lack of Al in the SEM-EDS spectrum.

### Implications for exposure

The potential of the identified metals and ceramics to catalyze harmful VEA reactions or aerosolize is dependent on the internal device temperatures achieved in actual usage [5, 13, 15, 23]. Previous studies have reported typical 100–400°C wetted coil temperatures, depending on the device design, flow conditions, voltage, and measurement position relative to the coil [10, 11, 17–18]. Temperatures as high as 1,000°C have been measured for “dry” coils without any e-liquid, so efficient wicking is important for delivering liquid to the coil and maintaining target coil temperatures [10, 23]. User avoidance of low liquid levels also may result in lower temperatures and lower toxic exposures [17].

The origins and usage conditions of the four THC cartridges from investigations are self-reported or unknown, and their operating temperatures could not be tested due to the previous extraction of their liquids. Nevertheless, the fact that both tested EVALI patient cartridges exhibited severe burn marks confirms that these cartridges were likely operated at high temperatures. This information could be used to help inform harm reduction efforts. In addition, knowledge of the materials in these four investigation-derived THC cartridges is potentially useful in the event that similar cartridges are used at high temperatures.

The combination of metals and ceramics identified in the newer, 2019 THC devices is consistent with conditions hypothesized for the formation of ketenes from VEA when sufficiently high temperatures are present [5–8]. The ceramic heating elements that were observed to fully occupy the cylindrical volumes additionally may act as insulators and promote higher temperatures. The microscopic pores in these ceramics enable diffusion of THC liquid throughout the interior of the heated ceramics. Due to the high specific surface area of this geometry, any catalytic interaction between the ceramic and THC liquid may be enhanced.

The Ni, Cr, Zn, Cu, Pb, Au, and Sn identified in these devices are potential sources of vaping exposures if heated enough to volatilize. Except for Au, these metals are consistent with those reported in previous nicotine device liquids and heated aerosol measurements [12–16]. These metals represent a potential chronic health risk when inhaled, though estimated risks have exceeded health limits mostly under high temperature or high usage scenarios [24, 25].

Several of these product designs expose polymer insulation, plugs, or sheaths directly to heat, especially in the THC cartridges. The wire sheaths and insulation fibers were < 5 mm and thus can be considered microplastics [26]. Studies from the literature suggest polymer thermal decomposition and volatilization may be a potential concern if internal temperatures reach 300°C or higher [27–29]. The polymers identified in this work were Si-rich rubbers, fluorinated polymers, and semi-synthetic rayon. FTIR of the rubber in one cartridge matched synthetic styrene-butadiene rubber (SBR) plus silica and calcite, two common rubber fillers [30]. Heating of SBR to 300–500°C can generate styrene and PAHs [27], though a laboratory study of a CCell THC device reported reduced emissions of styrene and other aromatic compounds compared to cannabis smoking [31]. It is also possible some of the other Si-rich rubbers may be silicones instead. Silicone compounds such as polydimethyl-siloxanes volatilize above 300°C [28], and more volatile silicone compounds are increasingly found as pollutants in indoor environments [32]. PTFE was identified by FTIR in one cartridge, a compound that can decompose and cause serious illness when heated above approximately 300 C [29]. There remains a possibility that the other fluorinated sheaths contain different perfluoroalkyls associated with a variety of chronic health effects [33]. Although comprehensive polymer testing is beyond the scope of this work, FTIR micro-spectroscopy has proven useful for microplastic identification (e.g., [34]), and could be pursued further for a more complete inventory of polymers.

### Metals characterization methods assessment

Portable XRF and SEM-EDS of the same nominal items were generally consistent but detected different elements in some cases, likely due to two main analytical differences: 1) The portable XRF did not detect lower-energy elements observed by SEM-EDS such as C and Si, and 2) The two techniques acquired data from different spatial scales. XRF signals were integrated across larger, centimeter-sized pieces of these products, while SEM-EDS focused on micrometer-sized regions from the disassembled components. For example, no Zr was detected by SEM-EDS in the EGO-CE6 filament or wick fibrils, so it is possible that the minor Zr detected by XRF was associated with heterogeneous regions of the wick not analyzed by SEM-EDS. Similarly, no Pb was detected in any of the individual battery contacts analyzed by SEM-EDS, so the minor Pb detected by XRF may have been in the metal end caps containing the battery contacts, which were too large to be included feasibly in the SEM-EDS analyses. In general, portable XRF was most useful for rapid scanning of heavy metals in the larger cartridge pieces, while SEM-EDS provided more detailed mineral, polymer, and metal compositions of their sub-components.

## Conclusions

The heavy metals identified in these devices are potential sources of chronic vaping exposures, though their ubiquitous presence in all tested products suggests that the metal components do not explain the relatively recent appearance of acute respiratory cases. It is possible that newer cartridges containing VEA may interact differently with these metals, especially if heated to higher temperatures. Notably, the newer cartridges associated with EVALI cases possessed the most insulating ceramic and polymer materials, and also exhibited more evidence of heat and thermal degradation than a used nicotine pod. These component compositions and high temperatures are consistent with conditions hypothesized to be sufficient for the formation of harmful chemicals (ethenone) from VEA. The fluorinated polymer components observed in some devices have the potential to thermally degrade and volatilize when heated. The apparent substitution of materials to achieve the same design goals, both between and within devices from the same vendors, suggests that the exact compositions of these components will continue to change over time.

This work is limited by the fact that many of the tested devices were samples from real investigations with unknown origins and usage parameters. The observed burn marks inside THC cartridges, potential for catalysis, and metal and polymer compositions do not imply that exposures would occur under all usage conditions, and are most relevant to harm reduction efforts based on avoiding higher internal temperatures. Because this work represents a limited sample size, these data should be confirmed by future testing of other THC cartridge compositions, both historical and contemporary. More internal temperature testing of various THC product types is needed as a function of usage conditions, configurations, and liquid levels.
